# Role of Dietary Catalpol Supplementation in Regulating Reproductive Development of *Harmonia axyridis*

**DOI:** 10.3390/insects17010020

**Published:** 2025-12-23

**Authors:** Keting Zhao, Qintian Shen, Sijing Wan, Liya Chen, Shiyu Tao, Yexin Xie, Min Zhou, Yan Li, Bin Tang

**Affiliations:** College of Life and Environmental Sciences, Hangzhou Normal University, Hangzhou 311121, China; 2023210301194@stu.hznu.edu.cn (K.Z.); 2023210301085@stu.hznu.edu.cn (Q.S.); wsjw9898@163.com (S.W.); liya1291803217@163.com (L.C.); 13858300864@163.com (S.T.); xyx7202023@126.com (Y.X.); zhoumin810611@126.com (M.Z.)

**Keywords:** *Harmonia axyridis*, artificial diets, catalpol, trehalase, reproductive development, energy metabolism

## Abstract

The mass rearing of natural enemy insects is a cornerstone of effective green pest control, making the development of optimized artificial diets critical for sustaining large populations. However, existing artificial diets often have limitations, including inhibitory effects on the reproductive development of natural enemies. In this study, we innovatively incorporated 20% catalpol into the artificial diets for *Harmonia axyridis*, aiming to comprehensively assess the effects of such dietary regimens on larval development, reproductive performance, and the regulation of energy metabolism in *H. axyridis*. Our results demonstrated that catalpol supplementation significantly shortened the duration of the 4th instar larval and pupal stages and increased pupal weight. Furthermore, catalpol treatment enhanced reproductive capacity, as evidenced by increased fecundity, improved hatching rate stability, and enhanced ovarian development. This effect was directly correlated with the significant upregulation of the vitellogenin genes *Vg1* and *Vg2* and their receptor gene *VgR* at 3 days post-emergence. Regarding energy metabolism, catalpol supplementation led to increased glycogen reserves and glucose content. The expression of the membrane-bound trehalase enzyme gene *TRE2* was significantly higher than that in the control group, and the expression level of the soluble trehalase enzyme gene *TRE1* also showed a significant increase. These findings indicate that catalpol effectively optimized energy metabolism, thereby accelerating developmental processes and enhancing reproductive capacity. In this study, we elucidated the mechanism by which catalpol regulates physiological metabolism in predatory natural enemy insects. For the first time, catalpol was incorporated into the artificial diet of *H. axyridis* as a synergistic component, significantly enhancing its growth, reproductive capacity, and energy metabolism. These findings provide a scientific basis for developing efficient artificial feeding systems and improving the field adaptability of natural enemy insects.

## Simple Summary

The large-scale reproduction of natural enemy insects is the prerequisite for achieving green control, and the development of artificial diets is the key to the expansion of natural enemy populations. This study investigated the growth and development, reproductive capacity, and energy metabolism levels of *Harmonia axyridis* under different feeding conditions and found that artificial diets supplemented with catalpol significantly promoted various physiological indicators of *H. axyridis*, indicating that catalpol has the potential to improve artificial diets for natural enemy insects and can be used in the industrialized cultivation of natural enemy insects and pest control.

## 1. Introduction

With the global population growth and the increasing demand for food, the threat of pests to crop yields is becoming increasingly severe [1,2,3,4]. The current pest control methods relying on chemical pesticides not only endanger the safety of agricultural products and ecological health but also hinder the sustainable development of agriculture [5,6,7]. In contrast, biological control, which utilizes natural enemy insects to suppress pests, can reduce pesticide residues and environmental pollution and delay the development of pest resistance, making it a key approach for green agriculture [8,9,10,11]. Studies have shown that natural enemies such as ladybugs, lacewings, and predatory mites have demonstrated good effects in the control of pests like aphids and mites [12,13,14]. However, achieving large-scale propagation of natural enemy insects remains the main bottleneck in promoting the industrialization of biological control [15], for which nutritional enhancement is an important direction for optimizing artificial feed and improving propagation efficiency.

The nutritional composition of food exerts a pivotal influence on the development and reproduction of insect species [16]. Research has demonstrated that an artificial diet for *Grapholita molesta*, primarily composed of wheat bran and soybean flour, significantly improves egg and pupal survival rates and egg hatchability, while reducing the incidence of adult morphological abnormalities [17]. The incorporation of 0.1% and 0.4% arabinoxylan into a semi-synthetic diet promotes the growth and development of fifth-instar silkworms, with the 0.1% supplementation also enhancing cocoon shell and pupal weight [18]. Studies on artificial diets for *H. axyridis* confirm that β-carotene supplementation significantly enhances larval survival and predation efficiency [19], and adding vitellogenin can improve their reproductive capacity [20]. Similarly, increasing shrimp meal content shortens the larval development period and improves survival rates, while elevated pollen content boosts larval survival and pupation success [15]. However, natural enemies reared on artificial diets often exhibit reduced survival rates, delayed development, diminished fecundity, and emergence abnormalities compared to their natural prey-reared counterparts [21]. For example, when *H. axyridis* was fed artificial diets based on pig liver, the larval development period was prolonged and adult fecundity was reduced [22].

Catalpol, a cyclic ether terpene glycoside extracted from the rhizome of *Rehmannia glutinosa* [23], exhibits various biological effects, including anti-inflammatory, antioxidant, hepatoprotective, and anti-diabetic properties [24,25]. Mammalian studies show that catalpol ameliorates triptolide (TP)-induced hepatotoxicity in murine models and AML12 hepatocytes, while mitigating glucose metabolism dysregulation and oxidative stress caused by mitochondrial dysfunction [26]. It also shows potential for clinical applications in treating oxidative stress and cell damage [27], and acts as a neuroprotective agent to facilitate post-stroke neurological recovery and ameliorate depressive phenotypes [28,29], possibly by inhibiting apoptotic pathways and activating the Nrf2/HO-1 signaling axis to mediate antioxidant responses [30]. Relevant studies have shown that catalpol, as a secondary metabolite of plants, can reduce the pathogen levels in bumble bees that consume food containing this component [31]. Furthermore, in the study on the selection of host plants for *Euphydryas anicia* larvae, it was found that although both of its two host plants, *Castilleja integra* and *Penstemon glaber*, contained catalpol, the content of catalpol in the latter was significantly higher. Correspondingly, the larvae also exhibit a faster growth rate and a higher survival rate on *P. glaber* [32]. Although the molecular mechanisms involved remain to be clarified, this phenomenon suggests that catalpol content may have a positive regulatory effect on the growth and development of insects. However, the regulatory mechanism of catalpol on the reproductive development of natural enemy insects remains entirely unexplored, and there is no report on the use of catalpol as a synergistic ingredient in the artificial diet of natural enemy insects.

*Harmonia axyridis* is a key predatory biocontrol agent against various Hemipteran and Lepidopteran pests [33,34]. Its 4th instar larvae and adults exhibit exceptional predatory capacity: adults consume an average of 288.97 nymphs of *Rhopalosiphum nymphae* (Linnaeus) per day [35]. The presence of *H. axyridis* is also associated with wing deformities, reduced reproductive capacity, and decreased survival rates in cotton bollworms (*Helicoverpa armigera* Hübner) [36]. Beyond direct predation, *H. axyridis* exerts non-consumptive effects on *S. frugiperda* larvae, inhibiting growth by prolonging pupal development, altering antioxidant enzyme activities, and disrupting nutrient assimilation [37]. It also exploits the sex pheromones of *S. frugiperda* for habitat location [38]. These findings underscore the profound impact and potential of *H. axyridis* in biological pest control, whose utilization can significantly reduce reliance on chemical pesticides.

For insects, yolk formation is an important process in population reproduction, involving the synthesis and absorption of vitellogenin (Vg), which serves as the precursor of vitellin (Vn) and provides essential nutrients for embryo development, such as amino acids, fats, vitamins, phosphates, and other trace elements [39,40,41]. In most insects, Vg is mainly synthesized in the fat body and secreted into the hemolymph and then absorbed by developing oocytes through endocytosis mediated by vitellogenin receptors (VgR). Therefore, Vg and VgR play crucial roles in insect reproduction [39,40]. As a key regulatory gene for vitellogenin synthesis, the expression level of the *Vg* gene is positively correlated with the reproductive capacity of insects. At the energy metabolism level, trehalose is the primary circulating sugar in insects, playing key roles in growth, stress protection, and recovery [42,43]. Trehalose metabolism is a critical regulatory pathway in insect physiology: it is synthesized by trehalose-6-phosphate synthase (TPS) during gluconeogenesis, transported to the hemolymph by trehalose transporters (TRET), and finally hydrolyzed into glucose by trehalase (TREH) [44]. Among them, TRE1 is a cytoplasmic enzyme used for hydrolyzing endogenous trehalose, while TRE2 is a transmembrane enzyme with its active site located on the outer side of the cell membrane, mainly hydrolyzing extracellular trehalose. Both can regulate and maintain trehalose homeostasis during the development and physiological processes of insects [45]. The *TRE1* and *TRE2* gene families contain multiple members. Among them, the *TRE1* family has identified *TRE1-1*, *TRE1-2*, *TRE1-3*, *TRE1-4*, *TRE1-5*, while the *TRE2* family includes *TRE2-1* and *TRE2-2*, which belong to different branches of the same gene family and share similarities in function or domain structure. Glycogen, trehalose, and glucose are mutually convertible in insects [46].

This study investigates the effects of dietary catalpol supplementation on larval development, reproductive performance, and energy metabolism in *H. axyridis*. By analyzing key physiological and molecular markers, we aim to elucidate the regulatory mechanisms of catalpol. The insights gained are expected to provide a theoretical foundation for enhancing the field adaptability of natural enemies and overcoming mass-rearing bottlenecks.

## 2. Materials and Methods

### 2.1. Source and Rearing of Test Insects

The tested insects were *H. axyridis* raised in an artificial climate room (temperature 25 ± 1 °C, relative humidity 70% ± 5%, photoperiod 14L:10D). They were reared on pea aphids (*Megoura crassicauda*). After the adult oviposited, the newly hatched larvae with well-developed colonies were selected for preservation. Some of the newly hatched larvae were used for propagation and subculture, while others were fed with pea aphids and cultivated to the 3rd instar larvae (3L) before being fed with feed, and the feed was changed daily until the 4th instar larvae (4L) pupated.

### 2.2. Formulation and Preparation of Artificial Diets

Two artificial diet formulations were used (details in Table 1). For the basic artificial diet: fresh pig liver was rinsed and mashed into a paste; 3 g sucrose, 2 g yeast powder, 2 g vitamin C powder, and 3 g royal jelly were weighed and mixed in a container, followed by the addition of 40 g mashed pig liver. After adding 3 g of agar to 30 mL of ddH_2_O, it was heated in a microwave oven and stirred constantly with a glass rod until the liquid became clear. The mixture was thoroughly stirred, and agar was added to form a semi-fluid basic diet. The catalpol-supplemented improved diet, which involved 20% catalpol, was prepared by adding 20 mL of catalpol master mix to the basic diet.

The catalpol master mix was prepared as follows: 4 mg catalpol was dissolved in 16 μL of 2% dimethyl sulfoxide (DMSO), and the volume was adjusted to 80 mL with double-distilled water (ddH_2_O).

### 2.3. Statistics of Physiological Characteristic Indicators Related to the Growth and Development of H. axyridis

This study set up three types of feeding groups. (1) Aphid group: *H. axyridis* fed with aphids. (2) Control group: basic artificial diet. (3) Catalpol group: artificial diet supplemented with catalpol. Well-developed third instar *H. axyridis* larvae were fed separately in three ways, with 16 to 30 larvae per group, and three biological replicates were set up. The date and time at which each larva transitioned into the fourth instar, pupated, and emerged were meticulously recorded. The durations of the third instar, fourth instar, and pupal stages were subsequently calculated, followed by a statistical analysis of the developmental timeline. At the beginning of the third instar stage, the beginning of the fourth instar stage, and the early pupal stage, each group of *H. axyridis* was weighed using an electronic balance, and the changes in their body weight were statistically analyzed.

### 2.4. Assessment of the Reproductive Capacity of the H. axyridis

Newly emerged *H. axyridis* adults were paired (male:female = 1:1) and assigned to the three feeding groups (Aphid, Control, Catalpol), with 15 pairs per group. The number of eggs laid per female was recorded daily. For hatching rate determination, 100–200 eggs were randomly collected from each group’s rearing container, with three biological replicates per group, and egg hatching was monitored.

Additionally, female adults were randomly selected from each group on the 1st, 3rd, and 5th days post-emergence for ovarian dissection. Ovaries were photographed using a Leica EZ4 HD stereomicroscope (Wetzlar, Germany) and LAS EZ software, and ovarian development was graded following the standard described by Gao et al. (2021) [47].

### 2.5. Extraction of Total RNA and cDNA Synthesis

Female *H. axyridis* adults were randomly selected from each group on the 3rd and 5th days post-emergence, with 6 females per replicate and three biological replicates per group. Total RNA was extracted from samples using a TRIzol reagent kit (Thermo Fisher Scientific Inc., Waltham, MA, USA) (following the manufacturer’s instructions). RNA integrity was verified via 1% agarose gel electrophoresis, and RNA purity and concentration were measured using a NanoDrop™ 2000 micro-nucleic acid spectrophotometer (Thermo Fisher Scientific Inc., Waltham, MA, USA). After the RNA concentration determination was completed, the corresponding content of RNA was calculated according to the formula: “The amount of RNA = 1000/The concentration of the measured RNA”. First-strand cDNA was synthesized using the PrimeScript™ RT Reagent Kit with gDNA Eraser (TaKaRa, Kusatsu, Japan) in RNase-free 500 μL PCR tubes. All operations were performed on ice to prevent RNA degradation.

### 2.6. Reverse Transcription Quantitative PCR (RT-qPCR)

Target genes included vitellogenin genes (*Vg1*, GenBank: KU761584.1; *Vg2*, GenBank: KY794939.1), vitellogenin receptor gene (*VgR,* GenBank: KY032000.1), and the soluble trehalase gene family (*TRE1-1*, GenBank: HM056038; *TRE1-2*, GenBank: FJ501961; *TRE1-3,* GenBank: JX514372; *TRE1-4,* GenBank: KP318742; *TRE1-5*, GenBank: KX349223) and the membrane-bound trehalase gene family *(TRE2-1*, GenBank: KX349224; *TRE2-2*, GenBank: KX349225). Ribosomal protein 49 (*rp49*, GenBank: AB552923) was used as the internal reference gene [48].

Primers for target and reference genes were designed using Primer Premier 5 software (with parameters optimized for specificity), and sequences are listed in Table 2 (synthesized by Zhejiang Shangya Biotechnology Co., Ltd., Hangzhou, China).

The RT-qPCR reaction system was prepared as follows: excluding template cDNA, the master mix contained TB Green dye, ddH_2_O, and forward/reverse primers at a volume ratio of 5:3.6:0.2. For each reaction, 9 μL of the master mix and 1 μL of cDNA template were added to individual wells of a 96-well plate, followed by centrifugation at 4000 rpm for 5 min at 4 °C.

Reactions were performed on a Bio-RAD CFX96 real-time fluorescence quantitative PCR instrument (Hercules, CA, USA) with the following conditions: initial denaturation at 95 °C for 30 s; 35 cycles of denaturation at 95 °C for 5 s, annealing at 85 °C for 5 s, and extension (with fluorescence detection) at 60 °C for 30 s. Relative gene expression levels were calculated using the 2^−ΔΔCt^ method [49].

### 2.7. Determination of Sugar Content and Trehalase Enzyme Activity Related to H. axyridis

Female *H. axyridis* adults were randomly selected from each group on the 3rd and 5th days post-emergence, with 3 females per replicate and three biological replicates per group. Twelve sterilized 1.5 mL Eppendorf tubes were divided equally into three groups (corresponding to Aphid, Control, Catalpol). Adult *H. axyridis* were dissected to remove heads, midguts, elytra, and membranous wings; the remaining tissues were placed into tubes (3 adults per tube, 4 tubes per group). A total of 200 μL phosphate-buffered saline (PBS, pH 7.4) was added to each tube. Samples were homogenized using a fully automated rapid sample grinder (program: 55 Hz for 60 s, 5 s interval, repeated twice), followed by ultrasonic homogenization at 4 °C for 30 min.

Total glycogen content, glucose content and trehalase enzyme activity were all quantified using the Sigma glucose detection kit (following the manufacturer’s instructions). Briefly, 160 μL of the 1000× *g* supernatant was mixed with 32 μL of 0.1 U/mL pullulan enzyme and incubated at 40 °C for 4 h for the determination of total glycogen and glucose content. Another 60 μL of the ultracentrifugation supernatant and the precipitate suspension were taken, and 75 μL of 40 mM trehalose solution and 165 μL of PBS were added to each. They were incubated at 37 °C for 60 min and then at 100 °C for 5 min for the determination of trehalase enzyme activity. Finally, 130 μL of aliquot was taken from each mixture and transferred to a new tube. Then, 260 μL of glucose detection reagent was added, vortexed to mix well, and incubated at 37 °C for 30 min. The reaction was terminated by adding 260 μL of 12 N sulfuric acid. Then, 200 μL of aliquot was added to each well of the microplate (three double-wells were set up for each treatment). The absorbance for total glycogen and glucose content was measured at 540 nm, and the trehalase enzyme absorbance was measured at 630 nm. Trehalose was quantified by the anthrone method. Each treatment was subjected to triple-well detection, and the absorbance was immediately measured at 630 nm after cooling.

### 2.8. Determination of Protein Content

Protein concentration was quantified using the BCA Protein Assay Kit (P0012, Beyotime, Shanghai, China). BCA working solution was prepared by mixing Reagent A and Reagent B at a volume ratio of 50:1 (25 mL Reagent A + 0.5 mL Reagent B) and vortexed thoroughly. A total of 200 μL working solution was added to each well of a microplate, followed by 20 μL of sample or protein standard. Each treatment was assayed in triplicate. After mixing, the microplate was incubated at 37 °C for 30 min, and absorbance was measured at 562 nm using a microplate reader.

### 2.9. Data Analyses

Statistical analyses were performed using SPSS Statistics 20, and graphs were generated using GraphPad Prism 10.1.2. All data are presented as mean ± standard error (mean ± SE). Differences between groups were analyzed via Tukey’s method in a one-way ANOVA using IBM SPSS Statistics 20. Different letters in the graph indicated significant differences between the two groups (*p* < 0.05).

## 3. Results

### 3.1. The Effect of Feeding Catalpol on the Weight and Developmental Duration of H. axyridis

**Figure 1:**
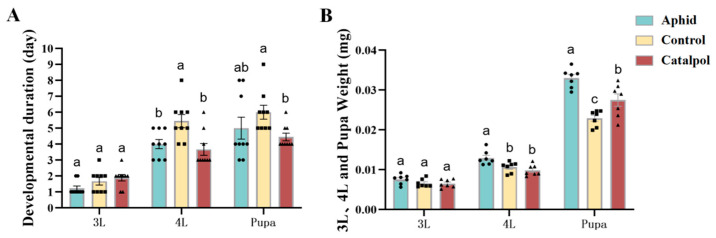
Effect of catalpol on the growth and development of *H. axyridis*. Groups Aphid, Control and Catalpol each contained 16 to 30 larvae and performed three biological repetitions. The development duration (**A**) and weight (**B**) of *H. axyridis* at various instars after being fed with aphids, basic artificial diets, and catalpol-supplement artificial diets. Significance analysis of the difference is conducted using Tukey’s test. Values are presented as the means ± SE. Different letters indicated significant differences between treatments (*p* < 0.05). Notes: Aphid: aphid feeding treatment; Control: control group; Catalpol: catalpol group (for ease of distinction, the aphid group, which was also the control group, was marked as aphid, and the basic artificial diet was marked as control).

Continuous monitoring and recording of the developmental stage of *H. axyridis* revealed that feeding *H. axyridis* with different diets exerted distinct impacts on its growth and development. The findings indicated that no significant differences were observed in the duration of the third instar larval stage of *H. axyridis* among different feeding treatments. The duration of the fourth larval stage and the pupal stage in the catalpol group was significantly shorter than that in the control group. However, there was no significant difference in the duration of the fourth larval stage and the pupal stage compared to the aphid group (Figure 1A). In the early third instar stage, no significant differences were observed in the effects of various food sources on body weight. During the early fourth instar stage, the body weight of the catalpol group was significantly lower than that of the aphid group, but showed no significant difference compared to the control group. In the early pupal stage (24 h post-pupation), the pupal weight of the catalpol group was significantly lower than that of the aphid group, yet significantly higher than that of the control group (Figure 1B). These results demonstrated that catalpol significantly shortens the developmental duration of *H. axyridis*, particularly for the 4th instar larval and pupal stages.

### 3.2. Effects of Feeding Catalpol on the Fecundity, Egg Hatching Rate and Ovarian Development of Female H. axyridis

**Figure 2:**
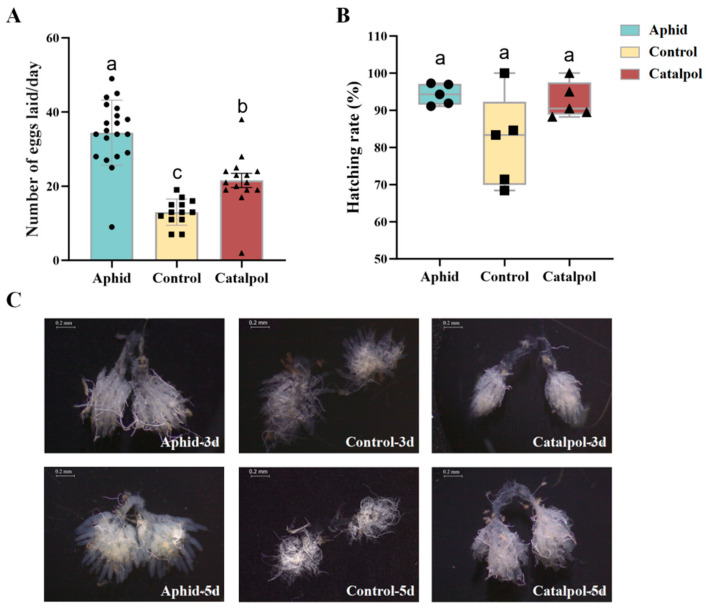
Effect of catalpol on the reproductive capacity of female *H. axyridis*. Groups aphid, control and catalpol each contained 15 pairs of *H. axyridis*. The number of eggs laid (**A**) and the hatchability (**B**) by each group was measured and calculated every day. Ovarian development images (**C**) were photographed by dissecting the ovaries of female adults on the 1st, 3rd, and 5th days. The significance analysis of the difference is conducted using Tukey’s test. Values are presented as the means ± SE. Different letters indicate significant differences between treatments (*p* < 0.05).

Compared with the control group, the number of eggs laid by the catalpol group and aphid group was significantly higher (Figure 2A). There was no significant difference in the hatching rate of *H. axyridis* eggs among different feeding methods (*p* = 0.109), and the hatching rate of all groups remained at a high range. However, the values of the control group had a wide range of distribution, a large degree of dispersion, and an obvious fluctuation in hatching rate, while the catalpol group had more concentrated data and more stable performance compared with the control group, which was similar to the performance of the ideal aphid group (Figure 2B). The ovarian development of the aphid group and the catalpol group was better than that of the control group. The ovarian ducts were enlarged and tubular, with yolk deposition and oocytes. However, no yolk deposition was observed in the control group, and the ovarian ducts were thin and transparent, with delayed ovarian development (Figure 2C). These results indicated that catalpol could promote ovarian development, yolk formation and egg hatching, increase the number of eggs laid, and then improve the fecundity of *H. axyridis*.

### 3.3. Effects of Feeding Catalpol on the Expression of Vg and VgR Genes in Female H. axyridis

**Figure 3:**
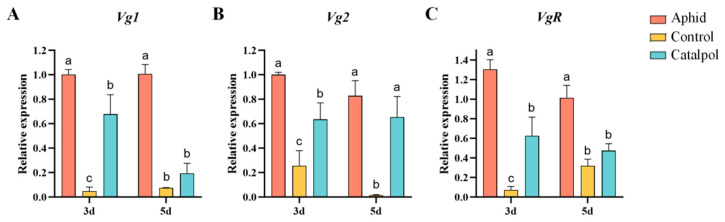
Effect of catalpol on the relative expression levels of reproduction-related genes in female *H. axyridis*. The expression levels of genes *Vg1* (**A**), *Vg2* (**B**) and *VgR* (**C**) in each group of female adults were detected by sampling on the 3rd and 5th days after being fed with aphids, basic artificial diets, and catalpol-supplement artificial diets. The significance analysis of the difference is conducted using Tukey’s test. Values are presented as the means ± SE. Different letters indicate significant differences between treatments (*p* < 0.05).

Quantitative analysis of reproduction-related gene expression in *H. axyridis* subjected to three dietary treatments revealed significant temporal variation. Under the 3-day feeding condition, the relative expression levels of *Vg1*, *Vg2* and *VgR* genes in the aphid group were significantly higher than those in the catalpol group, and the expression level in the catalpol group was significantly higher than that in the control group (Figure 3). When feeding was extended to 5 days, the expression levels of each gene in the aphid group remained at the highest level, but the expression differences of Vg1 and *VgR* genes between the catalpol group and the control group disappeared at this time (Figure 3A,C), while the *Vg2* gene was still significantly higher in the catalpol group than in the control group (Figure 3B). The research results showed that catalpol can up-regulate the expression levels of *Vg* and *VgR* genes in female *H. axyridis*.

### 3.4. Effect of Feeding Catalpol on the Content of Related Sugar and the Activity of Trehalase in H. axyridis

**Figure 4:**
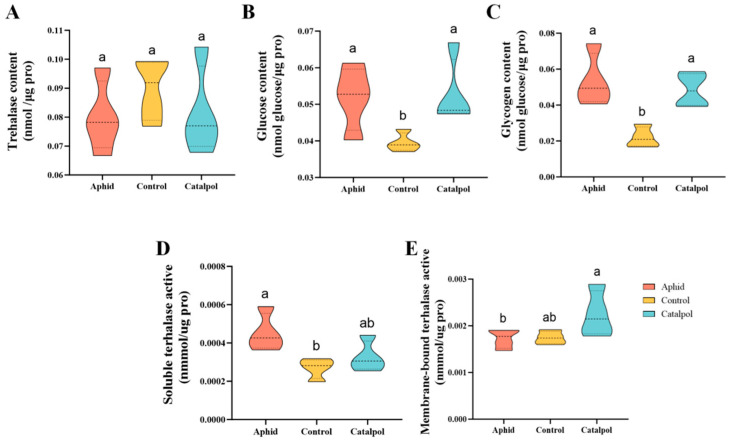
Effect of catalpol on the sugar content and enzyme activity of *H. axyridis*. The results were obtained by determining trehalose content (**A**), glucose content (**B**), glycogen content (**C**), soluble trehalase activity (**D**), and membrane-bound trehalase activity (**E**). The significance analysis of the difference is conducted using Tukey’s test. Values are presented as the means ± SE. Different letters indicate significant differences between treatments (*p* < 0.05).

In terms of substances related to energy metabolism in *H. axyridis*, there was no significant difference in glycogen and glucose contents between the aphid group and the catalpol group. Furthermore, the glycogen and glucose levels were found to be significantly higher than those of the control group (Figure 4B,C). However, there was no significant difference in trehalose content among the three treatment groups (Figure 4A). This indicates that, compared with basic artificial diets, catalpol can significantly increase the glycogen and glucose content in adult insects, with the same effect as aphids, but has no significant effect on trehalose content. The soluble trehalase enzyme (TRE1) activity in the catalpol group showed no significant difference from that in the aphid group, but it showed an upward trend compared with the control group (Figure 4D). However, the activity changes of the membrane-bound trehalase enzyme (TRE2) were different. The enzyme activity in the catalpol group was significantly higher than that in the aphid group, but there was no significant difference compared with the control group (Figure 4E). The research results showed that catalpol significantly increases the content of glucose and glycogen, as well as the activity of trehalase enzyme.

### 3.5. Effect of Feeding Catalpol on the Expression Levels of TRE1 and TRE2 Genes in H. axyridis

**Figure 5:**
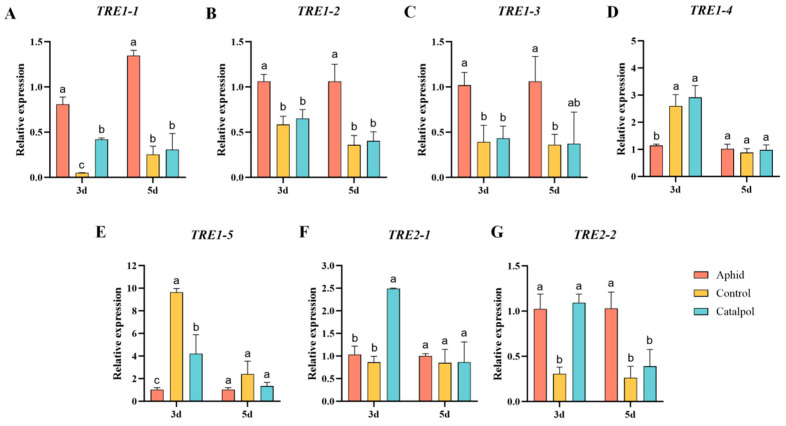
Effect of catalpol on the relative expression levels of enzymes related to energy metabolism in female *H. axyridis*. The expression levels of the *TRE1-1* (**A**), *TRE1-2* (**B**), *TRE1-3* (**C**), *TRE1-4* (**D**), *TRE1-5* (**E**), *TRE2-1* (**F**), and *TRE2-2* (**G**) genes were detected by sampling on the 3rd and 5th days after being fed with aphids, basic artificial diets, and catalpol-supplement artificial diets. The significance analysis of the difference is conducted using Tukey’s test. Values are presented as the means ± SE. Different letters indicate significant differences between treatments (*p* < 0.05).

By detecting the expression levels of energy metabolism-related genes in female *H. axyridis* under different treatments, it was found that after three days of feeding, *TRE1-1*, *TRE1-2*, and *TRE1-3* in the aphid group were significantly higher than those in the catalpol group and the control group. The gene expression levels in the catalpol group showed an upward trend compared with the control group. Moreover, the expression level of the *TRE1-1* gene was significantly higher than that in the control group (Figure 5A–C). The expression of *TRE1-4* and *TRE1-5* genes in the aphid group was significantly lower than that in the catalpol group and the control group. However, there was no significant difference in the expression level of TRE1-4 genes between the catalpol group and the control group. The expression level of *TRE1-5* genes in the control group was significantly higher than that in the catalpol group (Figure 5D,E). After 5 days of feeding, the expression levels of *TRE1-1* and *TRE1-2* genes in the catalpol group were significantly lower than those in the aphid group, and there was no significant difference compared with the control group (Figure 5A,B). In *TRE1-3*, the aphid group was significantly higher than the control group (Figure 5C), and there was no significant difference in the expression levels of *TRE1-4* and *TRE1-5* genes among the three groups (Figure 5D,E).

Meanwhile, catalpol differentially affected the expression of *TRE2* family genes. After 3 days of feeding, the expression level of the *TRE2-1* gene in the catalpol group was significantly higher than that in the aphid group and the control group (Figure 5F), and the expression of the *TRE2-2* gene was significantly higher than that in the control group, but there was no significant difference compared with the aphid group (Figure 5G). After 5 days of feeding, different feeding conditions had no significant effect on the expression of the *TRE2-1* gene (Figure 5F). The expression of the *TRE2-2* gene in the catalpol group was significantly lower than that in the aphid group, and there was no significant difference compared with the control group (Figure 5G). The results indicated that catalpol had a specific effect on the trehalase enzyme gene family, and its influence on membrane-bound trehalase enzyme *TRE2* gene was greater than that on soluble trehalase *TRE1* gene. Under the condition of feeding for 3 days, the gene expression level was significantly increased.

## 4. Discussion

Building on the well-documented positive effects of catalpol on glucose metabolism and oxidative stress in mammals [25], this study pioneers the application of catalpol as a dietary enhancer in artificial diets for natural enemy insects, focusing on its regulatory mechanisms for reproduction and development. The observation that the catalpol group exhibited shortened pupal duration, accelerated growth, and increased pupal weight (Figure 1A,B) aligns with findings by Li et al. (2020) [50], who showed that supplementing *H. axyridis* diets with energy substrates (e.g., glucose or trehalose) significantly enhances growth and development. Insects lose substantial water and desiccate during critical developmental stages due to their long development time, making the pupal stage a key window for developmental regulation [51]. We hypothesize that catalpol optimizes energy allocation in *H. axyridis* by modulating key metabolic pathways, thereby accelerating development and increasing body mass, providing a theoretical basis for refining artificial diets for natural enemy insects.

The efficacy of catalpol in improving ovarian function has been extensively validated in mammalian models [52,53,54]. In this study, the catalpol group showed higher cumulative egg production and more stable hatchability (Figure 2A), with improved ovarian development (Figure 2C), consistent with mammalian studies showing that catalpol protects and regulates ovarian development by modulating oxidative stress and apoptosis [55,56]. Although most catalpol research focuses on mammals, this study demonstrates similar synergistic effects in insects, confirming the feasibility of catalpol as a synergistic additive in artificial diets for natural enemy insects.

In insects, vitellogenesis is a fundamental process governing population reproduction, centered on the synthesis and uptake of vitellogenin (Vg) [46]. In this study, Vg1 and Vg2 transcription in the fat body of female *H. axyridis* was significantly upregulated in the catalpol group (Figure 3A,B), providing direct molecular evidence that catalpol activates vitellogenin synthesis at the transcriptional level, thereby promoting ovarian development and increasing egg production. This aligns with previous studies showing that basic artificial diets inhibit ovarian development and reduce *VgR* gene expression in *H. axyridis* [57], consistent with the control group results here. Thus, catalpol effectively overcomes this inhibitory effect and significantly enhances reproductive potential.

Previous studies have also shown that adding synergistic components to artificial insect diets significantly increases *Vg* expression [58]. Given that catalpol exerts similar effects, its value as a feed additive to enhance gene expression deserves further exploration. Additionally, juvenile hormone (JH) and ecdysis-triggering hormone (ETH) regulate insect behavioral plasticity and play pivotal roles in key life-history traits [59,60], while the ecdysone receptor (EcR) is critical for reproductive organ and oocyte development in *Coccinella septempunctata* (L.) [61]. We therefore hypothesize that catalpol may upregulate *Vg* and *VgR* expression by activating JH and ecdysone signaling pathways, a mechanism requiring further experimental validation. Although the catalpol group showed significant improvements, *Vg* and *VgR* expression remained lower than in the aphid group. Future research could optimize catalpol concentration, integrate complementary nutrients, or refine dietary composition to further enhance efficacy.

In this study, catalpol significantly increased glycogen and glucose contents in *H. axyridis* compared with the control group (Figure 4B,C), indicating enhanced glycogen reserves and glucose metabolism. No significant difference in trehalose content was observed among groups (Figure 4A), possibly because trehalose levels are regulated by multiple enzymes and sugar metabolism pathways [62], and the increase in trehalase activity may have hydrolyzed excess trehalose into glucose, resulting in stable trehalose levels.

Trehalose is an important energy storage substance during insect reproduction [63]. Catalpol may promote trehalose synthesis and accumulation in *H. axyridis* and increase trehalase substrate concentration by enhancing glucose utilization [64], thereby indirectly promoting trehalase activity and providing more energy for reproductive development [65]. Membrane-bound trehalase (TRE2) participates in insect energy metabolism and continuously supplies energy for growth and development [45]. In this study, there was no significant difference in TRE1 activity between the catalpol group and the aphid group, while the activity of TRE2 was significantly higher than that of the aphid group (Figure 4D,E). From an energy metabolism perspective, trehalase provides essential energy substrates and carbon skeletons for insect development and reproduction by hydrolyzing trehalose into glucose. This mechanism is supported by Wang et al. (2022) [66], who used RNA interference to silence *TRE1* and *TRE2*, resulting in reduced trehalase activity and significantly decreased egg fertilization, oviposition, and hatchability. In contrast, this study shows that catalpol upregulates trehalase activity, thereby promoting growth, development, and reproduction, confirming that catalpol modulates trehalase activity and enhances energy metabolism in *H. axyridis*.

Zheng et al. (2024) [67] also found that catalpol increases ATP synthesis and mitigates energy metabolism disorders in mice, whereas Li et al. [68] reported that catalpol improves mitochondrial dysfunction and increases ATP synthesis in diabetic mice. Based on these findings, we hypothesize that catalpol enhances trehalase activity and promotes carbohydrate synthesis in *H. axyridis*, thereby facilitating the production of energy molecules (e.g., ATP). This mechanism may play a crucial role in regulating energy homeostasis and providing the energy required for larval development and reproductive success. This study also found that catalpol exerts gene-specific effects on different members of the trehalase gene family, with varying intensity (Figure 5), revealing its targeted role in the glucose metabolism pathway, and its promoting effect on expression is particularly concentrated on *TRE2* genes. This is similar to the research results of Qin et al. (2025) [69], the trehalose, glucose content and *HaTreT* expression were all controlled by *HaTreh2*. In addition, TRE2 activity in the catalpol group was significantly higher than in the aphid group, but showed that *TRE2* gene expression in the catalpol group was only significantly elevated at 3 days and showed no difference at 5 days (Figure 4E and Figure 5G). The activity of enzymes depends not only on the quantity of proteins but also on their post-translational modification status. We speculate that catalpol treatment can continuously enhance the catalytic efficiency of its unit enzyme molecules by activating specific signaling pathways and triggering post-translational modifications such as phosphorylation of TRE2 enzymes [69]. This means that even if the gene transcription drops back to the basic level on the fifth day, the overall enzyme activity can still be significantly higher than that of the aphid group because the enzyme protein is in a highly activated state. In addition, persistently high enzyme activity accelerates the breakdown of trehalose, which may lead to changes in the levels of downstream metabolites such as glucose. Studies show that high glucose levels can reduce the expression of some genes [70]. Therefore, glucose may negatively regulate the transcription of the *TRE2* gene through intracellular energy states or feedback signaling pathways, restoring it to the baseline level on the fifth day and establishing a new metabolic homeostasis. We speculate that this may be achieved by activating key regulatory nodes [71,72], but further research is needed to clarify how catalpol remodels the glucose metabolism network to support reproductive energy demands.

## 5. Conclusions

In conclusion, this study demonstrates that catalpol serves as an effective dietary enhancer in artificial diets for natural enemy insects. Through a systematic investigation of developmental phenotypes, physiological parameters, and molecular regulatory mechanisms in *H. axyridis*, we have comprehensively elucidated the biological functions of catalpol as a feed supplement. Compared to traditional management strategies addressing “window periods” for natural enemy insects, the catalpol supplementation approach offers significant advantages in cost-effectiveness and operational simplicity. This innovative methodology provides a transformative solution for overcoming technical bottlenecks in the mass rearing of natural enemy insects.

## Figures and Tables

**Table 1:** Specific ingredients of the artificial diet formula for *H. axyridis*.

	Basic Artificial Diet	Improved Artificial Diet
Fresh pork liver	40 g	40 g
Royal jelly	3 g	3 g
Vitamin C	2 g	2 g
Sucrose	3 g	3 g
Yeast powder	2 g	2 g
Agar	3 g	3 g
ddH_2_O	30 mL	30 mL
Catalpol master mix		20 mL

**Table 2:** Primer sequences for qRT-PCR of *H. axyridis*.

Gene Name	Primer Sequence (5′-3′)	
	Forward Primer	Reverse Primer
*rp49*	GCGATCGCTATGGAAAACTC	TACGATTTTGCATCAACAGT
*Vg1*	GCAACAGAGTCCGTGGTCTTT	GCTGCTTTCACCGTTCTTCAA
*Vg2*	CAATCAAAACTCAAGCAAGGAGA	GTCAAAAACTGGATGGACAACAA
*VgR*	TGTAGGAGGCGAAGCAATGAT	TGGGATGTGACAGGGAAATAA
*TRE1-1*	CTTCGCCAGTCAAATCGTCA	CCGTTTGGGACATTCCAGAT
*TRE1-2*	TGACAACTTCCAACCTGGTAATG	TTCCTTCGAGACATCTGGCTTA
*TRE1-3*	ACAGTCCCTCAGAATCTATCGTC	GGAGCCAAGTCTCAAGCTCATC
*TRE1-4*	TTACTGCCAGTTTGATGACCAT	CATTTCGCTAATCAGAAGACCCT
*TRE1-5*	TGATGATGAGGTACGACGAGA	GTAGCAAGGACCTAACAAACTG
*TRE2-l*	TTCCAGGTGGGAGATTCAGG	GGGATCAATGTAGGAGGCTGTG
*TRE2-2*	CAATCAGGGTGCTGTAATGTCG	CGTAGTTGGCTCATTCGTTTCC

## Data Availability

The original contributions presented in this study are included in the article. Further inquiries can be directed to the corresponding authors.
